# THE JOINT EFFECTS OF WORK AND INFORMAL CAREGIVING ON MENTAL WELL-BEING AMONG CHINESE MIDDLE- TO OLDER-AGED ADULTS

**DOI:** 10.1093/geroni/igae098.2128

**Published:** 2024-12-31

**Authors:** Jing Ye

**Affiliations:** Wuhan University, Wuhan, Hubei, China (People’s Republic)

## Abstract

Although the health implications of employment and caregiving are well-documented, less is known about the joint impact of work and caregiving on the well-being of older adults. This study contributes to filling this research gap by focusing on the well-being implications of combining work and informal caregiving for Chinese older adults 60 years of age and older. The study draws on two theoretical perspectives: role strain theory and role enhancement theory. While the role strain theory assumes lower life satisfaction and higher depressive symptoms due to the double burden of paid work and caregiving, the role enhancement theory predicts an increase in well-being through the accumulation of resources and role fulfillment. Using data from the China Health and Retirement Longitudinal Study (CHARLS 2011-2018), this study aims at investigating whether and how older age working caregivers experience different life satisfaction and depressive symptoms. Besides, the moderating effects of intensity, gender, residence, socioeconomic status, and social isolation level are estimated. Results from the random-effects model show that compared to those who do not work or provide informal care, low-intensity worker-caregivers report higher life satisfaction. Only female worker-caregivers experience higher depressive symptoms, particularly with long hours of work and caregiving. These results support both role enhancement and role strain theories and suggest the need for increased investment in formal care facilities to alleviate the care burdens among older adults.

